# A pH-triggered self-releasing humic acid hydrogel loaded with porcine interferon α/γ achieves anti-pseudorabies virus effects by oral administration

**DOI:** 10.1186/s13567-024-01411-w

**Published:** 2024-11-20

**Authors:** Maoyuan Sun, Yongli Shi, Baishi Lei, Wuchao Zhang, Jingjing Feng, Shenghu Ge, Wanzhe Yuan, Kuan Zhao

**Affiliations:** 1 College of Veterinary Medicine, Hebei Agricultural University, Baoding, China; 2 College of Pharmacy, Xinxiang Medical University, Xinxiang, China; 3 Hebei Mingzhu Biotechnology Co., Ltd., Xingtai, China

**Keywords:** rPoIFNα/γ, pH-sensitive hydrogels, PRV, oral drug delivery system

## Abstract

Interferon α (IFNα) and interferon γ (IFNγ) play pivotal roles in mediating crucial biological functions, including antiviral activity and immune regulation. However, the efficacy of monomeric IFN is limited, and its administration relies solely on injection. To address this issue, we successfully expressed and purified a recombinant porcine IFNα and IFNγ fusion protein (rPoIFNα/γ). Furthermore, we developed a pH-triggered humic acid hydrogel delivery system that effectively protects rPoIFNα/γ from gastric acid degradation, enhancing its oral bioavailability. Neither the humic acid hydrogel nor rPoIFNα/γ exhibited cytotoxic effects on porcine kidney-15 (PK-15) cells in vitro. The replication of vesicular stomatitis virus and pseudorabies virus (PRV) was effectively inhibited by rPoIFNα/γ, resulting in an antiviral activity of approximately 10^4^ U/mL. Scanning electron microscopy revealed that the humic acid hydrogel had a loose and porous honeycomb structure. The IFNα/γ@PAM^gel^ hydrogel effectively adsorbed rPoIFNα/γ, as confirmed by Fourier transform infrared spectroscopy analysis, demonstrating a favourable IFN-loading capacity. In vitro experiments revealed that IFNα/γ@PAM^gel^ swelled and released IFNα/γ rapidly at pH 7.4 but not at pH 1.2. The oral administration of IFNα/γ@PAM^gel^ in mice enhanced the proliferation and differentiation of CD4^+^ and CD8^+^ cells. Additionally, mice infected with PRV and treated with IFNα/γ@PAM^gel^ presented increased transcription levels of interferon-stimulated genes in the serum, reduced mortality rates, lower viral loads in various tissues, and decreased levels of organ damage. In conclusion, this study demonstrates that orally administered IFNα/γ@PAM^gel^ has antiviral and immunomodulatory effects, highlighting its potential as a therapeutic agent against PRV infection.

**Supplementary Information:**

The online version contains supplementary material available at 10.1186/s13567-024-01411-w.

## Introduction

Pseudorabies, also known as Aujeszky's disease, is a highly pathogenic infectious disease caused by the pseudorabies virus (PRV) [1]. PRV is an enveloped virus with a linear double-stranded DNA genome of approximately 143 kb that encodes more than 70 proteins [2]. PRV can infect a wide range of mammalian species, including pigs, sheep, dogs, and rodents. The only natural host for PRV is pigs, and infection in pregnant sows leads to reproductive disorders such as spontaneous abortion and stillbirth, resulting in significant economic losses in the pig industry [3, 4]. Since 2011, PRV variant strains have been identified in China and have resulted in vaccination failure [5, 6]. More importantly, studies have shown that PRV can be transmitted to humans and cause respiratory dysfunction, neurological disorders, and conjunctivitis [7, 8]. Therefore, PRV infection may be a threat to public and human health.

Interferons (IFNs) are pivotal in immune regulation and are crucial cytokines in the host antiviral defence mechanism [9]. Three distinct classes of IFNs have been identified: type I (IFNα, IFNβ, IFNε, IFNκ, and IFNω), type II (IFNγ), and type III (IFNλ) [10]. However, IFN-υ has recently been found in the vertebrate genome sequence. These findings suggest that type IV interferons may be present [11]. Among these IFNs, type I IFNs, particularly IFNα, demonstrate broad-spectrum antiviral activities against various viruses compared with other IFN types. IFNγ, the only type II IFN, is pivotal in innate and adaptive immunity against viral and intracellular bacterial infections [12–14]. Porcine IFN (PoIFN)-α and -γ have been extensively utilized in the treatment and prophylaxis of viral diseases. For example, they are employed to treat animal diseases caused by transmissible gastroenteritis viruses and African swine fever virus [15, 16]. However, several challenges persist in the utilization of IFN. For example, single-component IFNs lack the dual functionality of antiviral and immunomodulatory effects. Furthermore, the administration of protein drugs, primarily via injection, poses significant stress on animals and is difficult to perform, thereby severely restricting the utilization of IFNs.

Hydrogels are synthesized through covalent or noncovalent interactions between diverse polymeric materials and have been extensively investigated because their distinctive properties, including biocompatibility, permeability, and wettability, resemble those of human tissues [17–19]. Humus has been extensively utilized in the medical and veterinary fields because of its antiviral, antibacterial, and anti-inflammatory properties [20–22]. Humic acid (HA), a derivative of humus, is an organic substance generated through the decomposition of animals and plants. It constitutes approximately 50% of the Earth's natural organic carbon reservoirs, which are extensively distributed across terrestrial soils, rivers, lakes, and marine environments [23–25]. Because HA hydrogels exhibit ultralow systemic toxicity, they are also used as carriers for cancer treatment [26]. In recent years, researchers have discovered that incorporating hydrophobic moieties can increase the stability of hydrogels. HA molecules possess both hydrophobic and active functional groups, making them promising candidates for the fabrication of hydrogels [27–30]. Because HA is rich in adsorption sites, the adsorption properties of the hydrogels can be further enhanced [31]. Therefore, the utilization of HA for hydrogel modification represents a highly promising strategy. The natural proteinaceous macromolecule gelatin (Gel), derived from collagen denaturation as a byproduct of meat production, has been deemed safe by the US Food and Drug Administration (FDA) [32]. Gels are widely employed in the fabrication of synthetic hydrogels, and researchers have successfully integrated them with humus to yield 3D hydrogels that exhibit significantly enhanced bactericidal properties [33, 34]. The raw materials potassium humate (KHA) and Gel can be utilized along with acrylamide (AM), 2-acrylamido-2-methyl-1-propanesulfonic acid (AMPS), *N,N*′-methylene bisacrylamide (MBA), and potassium persulfate (KPS) to fabricate pH-sensitive HA hydrogels through aqueous polymerization.

**Figure 1:**
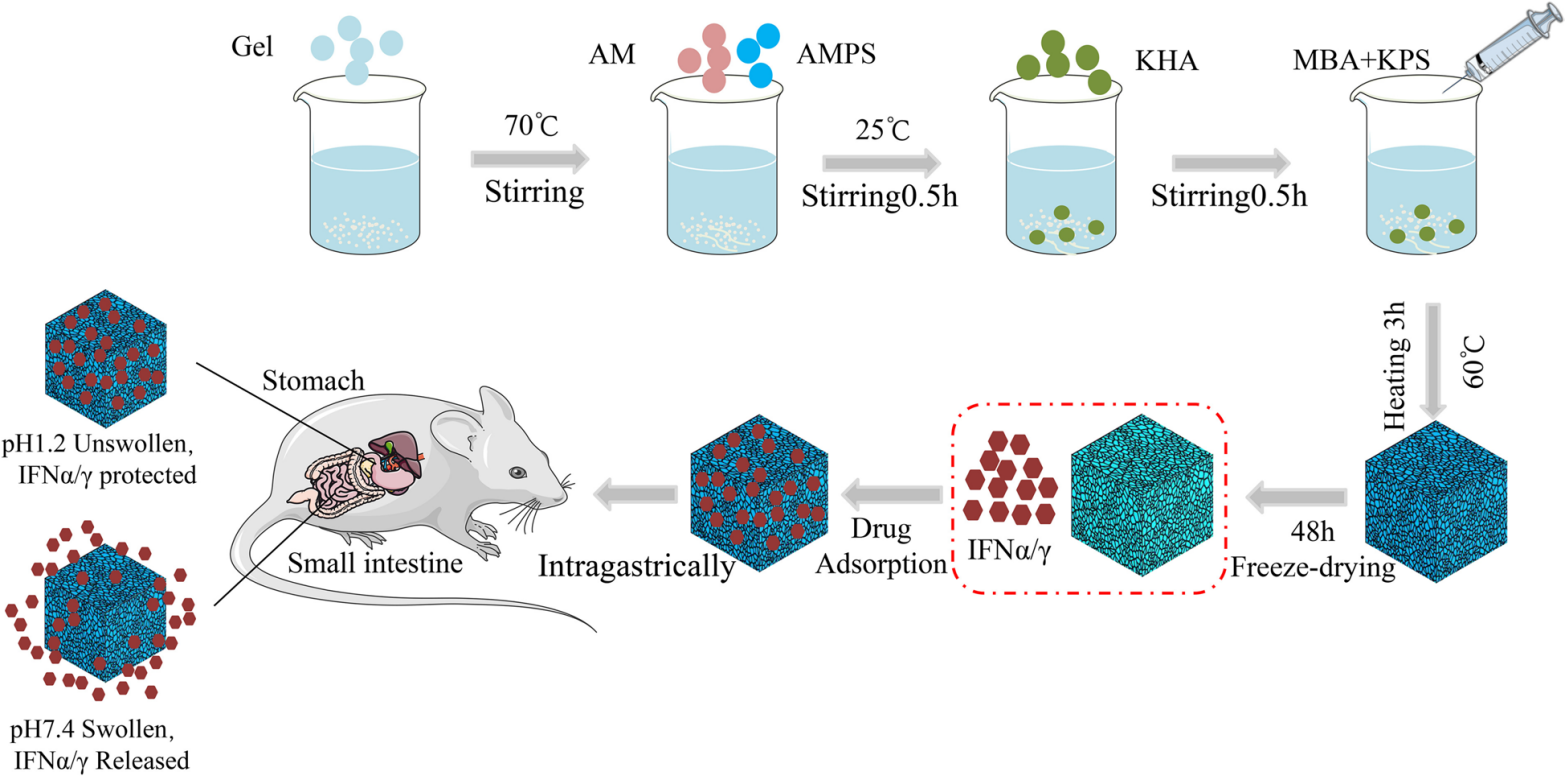
**Schematic representation of the rPoIFNα/γ release pattern at pH 1.2 (stomach) and pH 7.4 (intestine) and the hydrogel synthesis route**. rPoIFNα/γ, recombinant porcine interferon α and γ fusion protein.

The present study investigated the expression of IFNα and γ fusion protein (IFNα/γ) as a strategic approach to enhance the antiviral and immune regulatory efficacy of IFN. To enable the oral administration of IFNα/γ, we prepared HA hydrogels loaded with IFNα/γ (IFNα/γ@PAM^gel^) (Figure 1). The safety and efficacy of the generated IFNα/γ@PAM^gel^ were evaluated both in vitro and in vivo. These results will help promote the development of highly effective IFN drugs and oral delivery systems to help prevent and treat pseudorabies.

## Materials and methods

### Reagents, cells, viruses, and animals

The gelatin was procured from Sigma Biotechnology Co., Ltd. (USA). The chemicals AM (99% purity), KPS (99.5% purity), AMPS (98% purity), MBA (analytical grade), and KHA (70% purity) were purchased from Shanghai Macklin Biochemical Co., Ltd. (China). Anti-mouse CD4, FITC (Clone: GK1.5) and anti-mouse CD8α, PE (Clone: 53–6.7) antibodies were obtained from Hangzhou Multi Sciences Biotechnology Co., Ltd. (China). DH5α and BL21(DE3) bacteria were procured from Beijing Quanshi Gold Biotechnology Co., Ltd. (China). PK-15 and African green monkey kidney (Vero) cells were cultured in Dulbecco’s modified Eagle’s medium (DMEM) supplemented with 10% fetal bovine serum (Gibco, Thermo Fisher Scientific, Waltham, MA, USA). The cells were maintained at 37 °C in 5% CO_2_. The PRV variant strain HB1201 was isolated in our laboratory [35]. Six-week-old BALB/c mice were purchased from Beijing Huafukang Biotechnology Co., Ltd. (China).

### Expression and purification of recombinant PoIFNα/γ

The coding sequences for porcine IFNα (accession no. KF414740.1) and IFNγ (accession no. NM213948.1) were analysed and optimized with NanoPro and JCat. The signal peptide sequences were removed. The optimized sequence was connected using a flexible linker (GGGGSGGGGSGGGGS) that was synthesized and cloned and inserted into the pET28a vector. The rPoIFNα/γ protein was expressed and purified following previously established protocols, and the expression of recombinant proteins in the form of inclusion bodies is very common. The purified recombinant proteins can be made biologically active by refolding in refolding buffer at 4 ℃ for 48 h, which has been used in many other studies [36, 37]. The purity of the isolated rPoIFNα/γ was assessed by SDS‒PAGE and western blotting. The primary antibody used was a mouse anti-His mAb, and the secondary antibody used was horseradish peroxidase (HRP)-coupled goat anti-mouse IgG (Biodragon Biotechnology Co., Ltd., China). The concentration of purified rPoIFNα/γ was determined using a BCA protein assay kit, and the lipopolysaccharide in the recombinant protein was eliminated with an endotoxin removal kit. (Solarbio, Beijing, China), following the manufacturer's instructions. The purified rPoIFNα/γ was stored at -80 ℃ for long-term preservation.

### Determination of rPoIFNα/γ cytotoxicity

The cytotoxicity of rPoIFNα/γ was assessed using a Cell Counting Kit-8 (CCK-8) assay (Solarbio) following the manufacturer’s instructions. PK-15 cells were seeded in 96-well plates and grown to 90% confluence. PK-15 cells were used to evaluate the cytotoxicity of the drug. This is a common research method used in many other similar studies [38, 39]. In accordance with the fourfold dilution method, 300 μL of rPoIFNα/γ (0.8 mg/mL) was diluted with 900 μL of DMEM containing 2% foetal bovine serum, resulting in a total of seven dilution gradients; eight repetitions were used for each gradient. Subsequently, 100 μL of diluted protein was added to each well. Following a 24 h incubation period, 10 μL of CCK-8 was added, and the mixture was incubated at 37 ℃ for 2 h. The absorbance at 450 nm was subsequently measured using a microplate reader.

### Determination of the antiviral activity of rPoIFNα/γ in vitro

As previously described, the antiviral activities of rPoIFNα/γ were assessed using a cytopathic effect (CPE) inhibition assay involving vesicular stomatitis virus (VSV) and PRV in Vero and PK-15 cells [40]. Briefly, when the cells in the 96-well plates reached 90% confluence, they were treated with 100 μL of rPoIFNα/γ at fourfold serial dilutions for 16 h, followed by incubation with VSV or PRV (100 TCID_50_/well). Eight wells without virus were used as negative controls, and eight wells with virus but no rPoIFNα/γ were used as positive controls. The active titre of rPoIFNα/γ was determined after 48 h using the Reed–Muench method [41].

### Preparation of pH-sensitive hydrogels

pH-responsive hydrogels were synthesized via aqueous solution polymerization. AM, AMPS, MBA, and KPS were combined and dissolved in 10 mL of deionized water. Subsequently, 0.1 g of KHA was dissolved in 10 mL of deionized water and stirred well with the gel mixture for 30 min to achieve a homogeneous suspension, which was then incubated at 60 °C for 3 h. The resulting products were thoroughly rinsed with deionized water and ethanol three times to remove unreacted monomers and obtain purified hydrogels. The wet hydrogels were lyophilized using a SCIENTZ-10 N vacuum freeze drier (Ningbo SCIENTZ Science and Technology Corporation, China). The hydrogel without cargo was designated PAM^gel^. An aliquot of dry hydrogel was immersed in a solution of IFNα/γ at 4 °C for 12 h. The IFN-loaded hydrogel was designated IFNα/γ@PAM^gel^.

### Characterization of pH-sensitive hydrogels

The morphology and structural characteristics of the hydrogels were observed by scanning electron microscopy (SEM, Phenom Pro microscope, USA) with an accelerating voltage of 15 kV [42]. Fourier transform infrared spectroscopy (FT-IR) was performed on the hydrogels and interferon solutions via an FT-IR spectrometer (PerkinElmer, China). The samples were subjected to differential scanning calorimetry (DSC) analysis using a TA Instruments system at a scanning rate of 10 °C/min across a 50 °C–300 °C temperature range. The cytotoxicity of the hydrogels was assessed using established protocols as previously described [43, 44]. The swelling properties of the cargo-free PAM^gel^ were assessed using a previously established methodology [45].

### rPoIFNα/γ release studies

In vitro drug release studies were conducted by immersing an aliquot of the IFN-loaded hydrogel in simulated gastric fluid (pH 1.2) or intestinal fluid (pH 7.4). The mixture was incubated at 37 °C with rotational mixing at 100 rpm. At specific time intervals (0, 4, 8, 12, 16, 20, and 24 h), an aliquot (0.5 mL) was sampled for detection. An equal volume of fresh buffer solution was subsequently added to restore the initial volume. The release of IFN in the solution was determined by measuring the absorbance at 280 nm using UV/VIS spectrophotometry [46, 47].

### Intestinal safety and CD4^+^ and CD8^+^ T-cell assessments

A total of 24 6-week-old female BALB/c mice were randomly allocated into two groups: the IFNα/γ@PAM^gel^ treatment group and the phosphate-buffered saline (PBS) control group. The IFNα/γ@PAM^gel^ treatment group received an oral administration of IFN adsorbed by the hydrogel at a dose of 100 µg/mouse for three consecutive days, whereas the control group was administered an equivalent volume of PBS. The peripheral blood and spleen tissues of the mice in each experimental group were collected at specific time points (1, 3, 5, and 7 days) following drug administration. The levels of CD4^+^ and CD8^+^ T cells and interferon-stimulated gene (ISG) transcription were subsequently quantified. Each assay contained at least 100 µL of peripheral blood sample and was labelled with the corresponding antibodies. Immediately after sample preparation, flow cytometry (FCM) was used for prompt detection and analysis (Attune NxT, Thermo Fisher Scientific, USA) [48]. After the mice were treated for three days, the duodenum was fixed with a 10% formalin solution, followed by tissue section preparation and subsequent evaluation of intestinal safety.

### Quantitative reverse transcription polymerase chain reaction (qRT-PCR)

To evaluate the capacity of IFNα/γ@PAM^gel^ to induce ISG production in mice, the expression of the three ISGs in the spleen was analysed. Total RNA was extracted from the spleen tissue via TRIzol RNA extraction reagent (Takara Bio Inc., Japan). The Pkr, Ifit1, and Isg20 levels were quantified via TaqMan real-time PCR. Relative expression levels were determined via the 2^−ΔΔCt^ method. To standardize the values for each sample, β-actin mRNA was used as an internal control. The primers utilized for the ISGs are shown in Table 1.

**Table 1:** **Primers used for gene cloning and qPCR analysis**

Primers	Nucleotide sequence (5′ → 3′)
Mouse-PKR-F	ATGCACGGAGTAGCCATTACG
Mouse-PKR-R	TGACAATCCACCTTGTTTTCGT
Mouse-IFIT1-F	TACAGGCTGGAGTGTGCTGAGA
Mouse-IFIT1-R	CTCCACTTTCAGAGCCTTCGCA
Mouse-ISG20-F	CTTGGGCCTCAAAGGGTGAG
Mouse-ISG20-R	CGGGTCGGATGTACTTGTCA
Mouse-β-actin-F	CATTGCTGACAGGATGCAGAAGG
Mouse-β-actin-R	TGCTGGAAGGTGGACAGTGAGG
PRV-rtgB-F	GTCACCTTGTGGTTGTTG
PRV-rtgB-R	CCACATCTACTACAAGAACG

### Determination of anti-PRV activity in vivo

Thirty 6-week-old BALB/c female mice were randomly allocated to PRV infection, IFNα/γ@PAM^gel^ treatment, or PBS control groups. The challenge dose was determined on the basis of previously reported findings [49–52]. Mice in the PRV infection and IFNα/γ@PAM^gel^ treatment groups were intramuscularly infected with 200 μL of PRV at a titre of 10^2.5^ TCID_50_. The IFNα/γ@PAM^gel^ treatment group received an oral administration of interferon adsorbed by the hydrogel at a dose of 100 µg/mouse for three consecutive days, starting 12 h after the challenge. The PBS control group remained uninfected with PRV and did not receive treatment with IFNα/γ@PAM^gel^. All surviving mice were euthanized 14 days post-treatment (dpt), after which the spleen, lungs, and brain were collected. Tissues from the mice that died during the experiment were collected immediately after death. Viral DNA was extracted from tissue sections. The number of viral DNA copies was determined via qPCR [53]. The primers used in this study are shown in Table 1.

### Statistical analysis

The statistical analyses were conducted using GraphPad Prism 6 (GraphPad Software Inc., La Jolla, CA, USA). Statistical significance was assessed using Student’s *t* tests. A value of *p* < 0.05 indicated statistical significance.

## Results

### Expression and purification of rPoIFNα/γ

**Figure 2:**
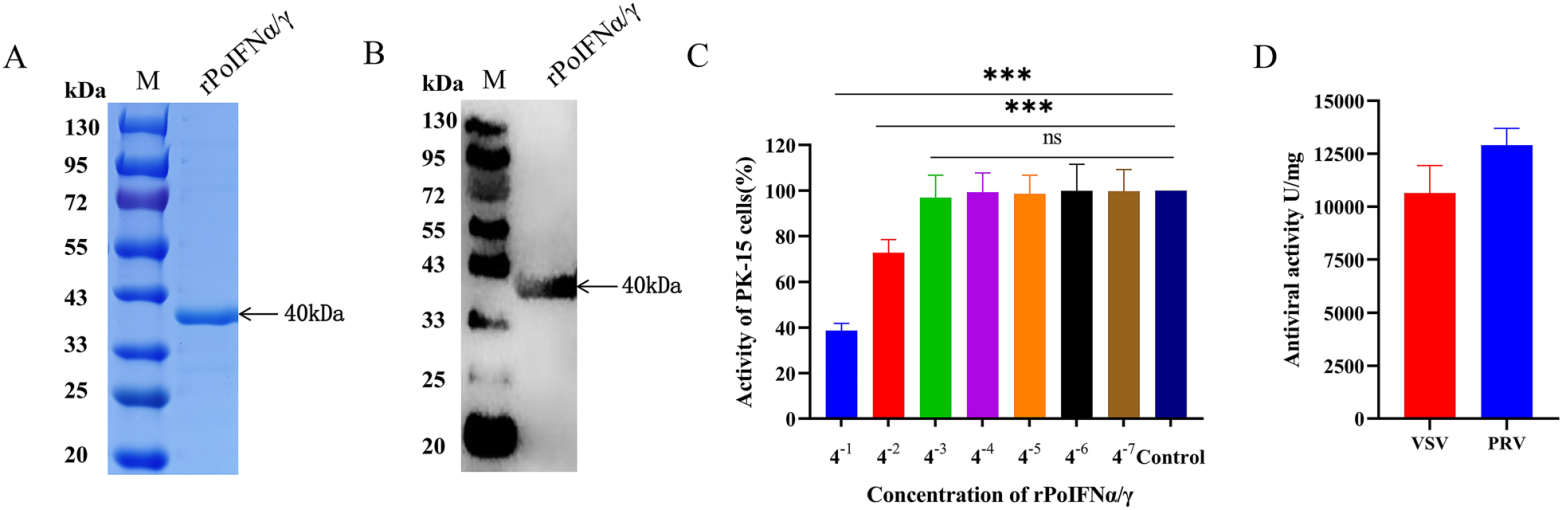
**Expression, purification, toxicity assessment, and antiviral activity evaluation of the rPoIFNα/γ recombinant protein. A** SDS‒PAGE analysis of the purified rPoIFNα/γ protein. **B** Western blot analysis of the rPoIFNα/γ protein. **C** Cytotoxicity of various concentrations of rPoIFNα/γ on PK-15 cells was assessed after 24 h of incubation. Statistical significance was determined using Student’s *t* tests (***p < 0.001; ns, not significant). **D** Antiviral activity of rPoIFNα/γ against vesicular stomatitis virus (VSV) and pseudorabies virus (PRV) was assessed in vitro using the cytopathic effect inhibition method.

The coding sequences of rPoIFNα and rPoIFNγ were optimized and linked using a flexible linker. The tandem fusion sequence of rPoIFNα and rPoIFNγ (rPoIFNα/γ) is presented in Additional file 1. The rPoIFNα/γ sequence was subsequently cloned and inserted into the pET28a vector, which was subsequently transformed into BL21(DE3) cells for expression. The findings of this study indicate that the expression of rPoIFNα/γ occurred in the form of inclusion bodies. The rPoIFNα/γ was purified with Ni-agarose resin. The SDS‒PAGE results revealed a major band with a molecular weight of 40 kDa (Figure 2A), which was verified to be tagged by western blotting with an anti-His antibody (Figure 2B). Thus, rPoIFNα/γ was expressed and purified at high concentrations for subsequent experimental use.

### Cytotoxicity and antiviral activity of rPoIFNα/γ in vitro

The cytotoxicity of rPoIFNα/γ was evaluated in PK-15 cells using the CCK-8 assay. Compared with that of untreated control cells, the viability of rPoIFNα/γ-treated PK-15 cells was significantly lower at dilutions ranging from 4^–1^ to 4^–2^ (Figure 2C). The viability of PK-15 cells remained unaffected by dilutions ranging from 4^–3^ to 4^–7^. These findings validate the safety profile of rPoIFNα/γ in PK-15 cells, establishing a foundation for subsequent investigations. The antiviral activities of rPoIFNα/γ were determined using the CPE inhibition method. PK-15 cells were incubated with dilutions of rPoIFNα/γ for 12 h and subsequently infected with either PRV or VSV. The results demonstrated that the antiviral activity of rPoIFNα/γ against VSV and PRV was approximately 10^4^ U (mg/mL) (Figure 2D). These findings demonstrated that rPoIFNα/γ effectively suppressed the replication of VSV and PRV in PK-15 cells.

### SEM and cytotoxicity assay of the hydrogels

**Figure 3:**
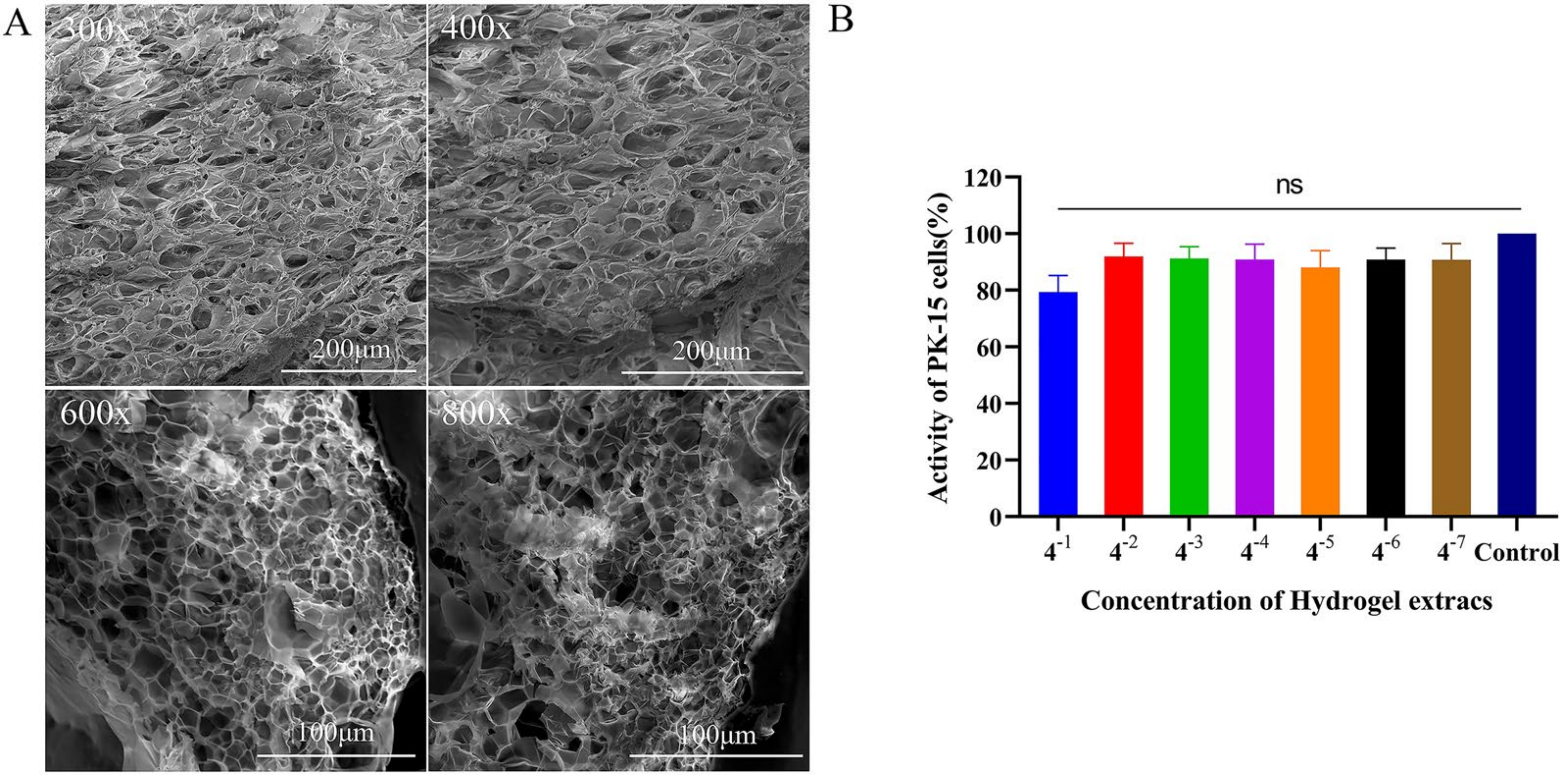
**SEM images at different magnifications and toxicity evaluation of hydrogels. A** SEM images of the hydrogels were captured at various magnifications, including × 300, × 400, × 600, and × 800. **B** Cytotoxicity of the hydrogel extracts at various concentrations against PK-15 cells after incubation for 24 h.

As shown in Figure 3A, the structural characteristics of the cargo-free PAM^gel^ were examined using SEM, revealing a honeycomb-like appearance at various magnifications (× 300, × 400, × 600, and × 800). These dispersed network structures can function as efficient drug carriers, offering robust drug loading and release support. After calculation, the porosity of the cargo-free PAM^gel^ was determined to be 60.1 ± 8.89% (Additional file 2). To further verify the safety of the hydrogels, the cytotoxicity of the cargo-free PAM^gel^ was determined using a cytotoxicity assay (CCK-8). The results revealed that after the cells were incubated with different concentrations of the extracts for 24 h, the cell viability was greater than 75% (Figure 3B). This level was classified as level 1 according to the corresponding evaluation criteria (Table 2). There was almost no toxicity to cells. These results preliminarily confirmed the safety of the hydrogels as drug carriers and provided a rationale for subsequent animal experiments.

**Table 2:** **Toxicity scores of the hydrogel extracts**

Score	RGR (%)
0	≥ 100
1	75 ~ 99
2	50 ~ 74
3	25 ~ 49
4	1 ~ 24

RGR = (OD of test group/OD of control group) × 100%.

### FT-IR and DSC analyses

**Figure 4:**
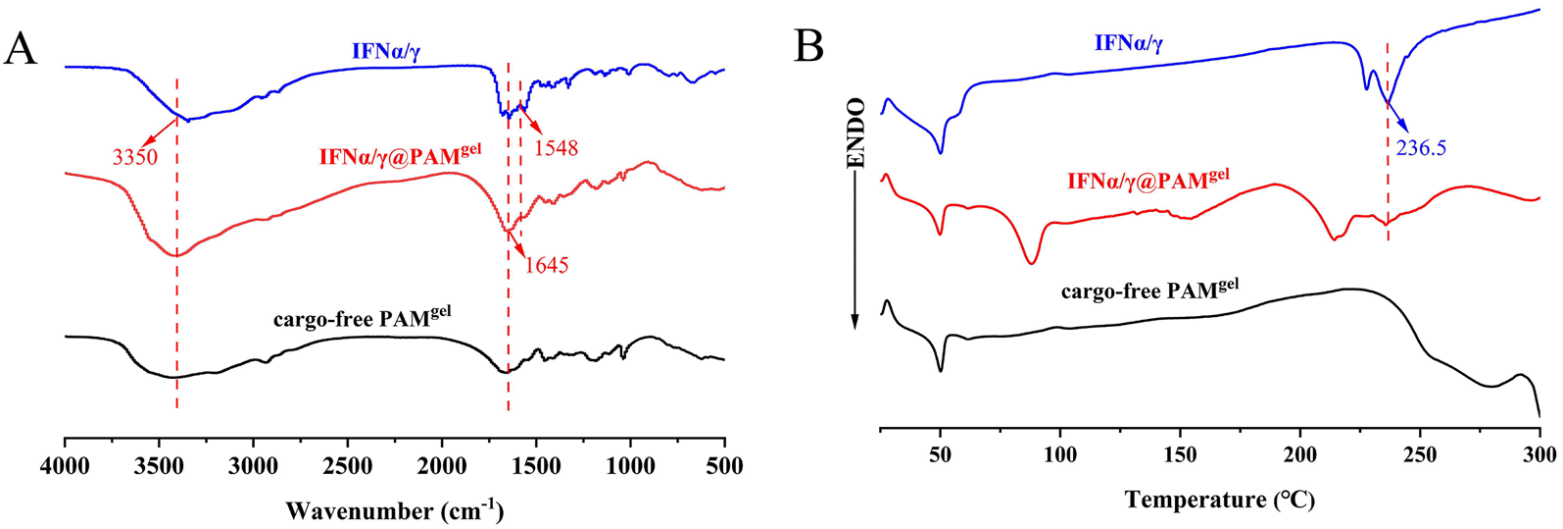
**FT-IR and DSC analysis of IFNα/γ, IFNα/γ@PAMgel, and PAM**^gel^. **A FT-IR spectra.** **B DSC spectra.**

FT-IR and DSC analyses are viable methods for investigating drug‒hydrogen atom interactions. In the FT-IR spectrum of the cargo-free PAM^gel^ (Figure 4A), the absorption bands observed at 3420 cm^−1^ and 1645 cm^−1^ may be caused by the stretching vibrations of the hydroxyl and carbonyl groups. In the amide II region, a distinct peak at 1548 cm^−1^ was observed for both IFNα/γ and IFNα/γ@PAM^gel^, indicating the presence of a natural helical structure and protein aggregates. Furthermore, the absence of this characteristic peak in the cargo-free PAM^gel^ confirms the successful loading of interferon onto the hydrogel. The distribution of IFNα/γ in the hydrogel was also investigated. The DSC patterns of IFNα/γ, cargo-free PAM^gel^, and IFNα/γ@PAM^gel^ are shown in Figure 4B. An endothermic peak was observed in the DSC pattern of free IFNα/γ, which was caused by the fusion of IFNα/γ crystals. In contrast, the observed peak in the DSC pattern of IFNα/γ@PAM^gel^ exhibited a noticeable decrease. This phenomenon suggested that most of the IFNα/γ distributed in IFNα/γ@PAM^gel^ had an amorphous or molecular state, which improved the bioavailability and release rates of the encapsulated IFNα/γ.

### Swelling behavior of hydrogels and IFNα/γ release studies

**Figure 5:**
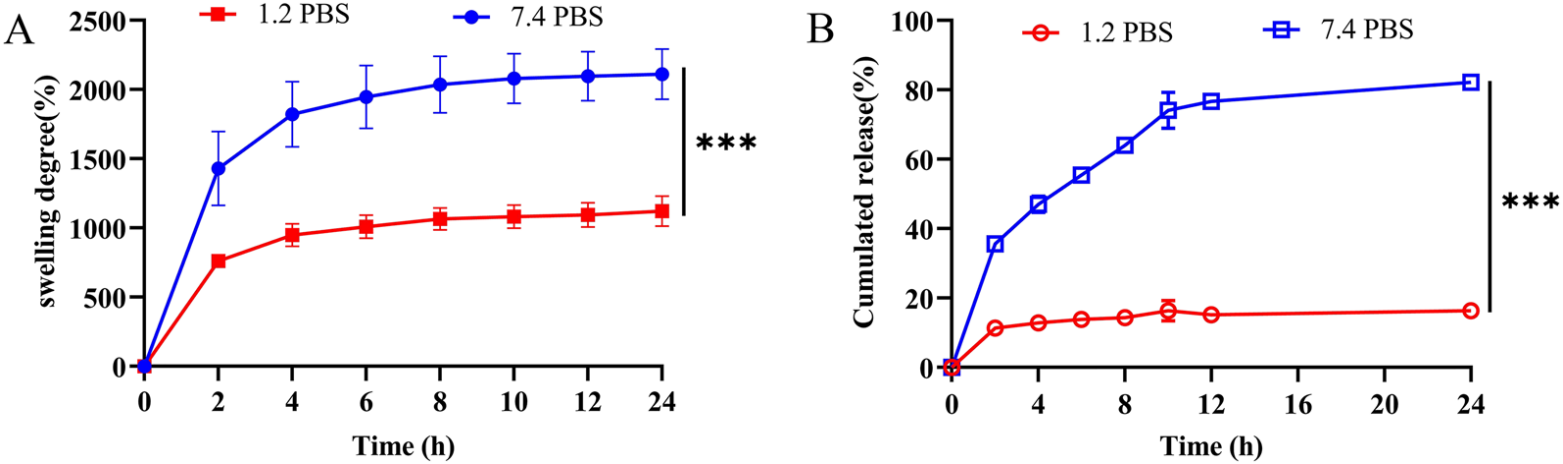
**Swelling behavior of hydrogels and cumulative drug release in buffers at different pH values. A** Swelling behavior of hydrogels in buffers at pH 1.2 and 7.4. **B** Cumulative release of rPoIFNα/γ from hydrogels in different pH conditions representing gastric fluid (pH 1.2) and intestinal fluid (pH 7.4) at 37 °C. The results are the mean ± standard deviation (*n* = 3).

To mimic the swelling behavior of hydrogels and the release of IFNα/γ in the gastric and intestinal environments, in vitro experiments were conducted at pH 1.2 and 7.4, respectively. After 24 h of immersion, the maximal swelling values were recorded as 1122.7 ± 109.7% (pH = 1.2 PBS) and 2112.1 ± 181.2% (pH = 7.4 PBS) (Figure 5A). These findings suggest enhanced delivery of drug molecules within the slightly alkaline environment (e.g., the intestinal tract). The results also revealed that the release of IFNα/γ from the hydrogel had a typical pH dependence. After 24 h of incubation, only 16.4 ± 0.1% of the IFNα/γ was released at pH 1.2 in PBS. In contrast, 82.3 ± 0.3% of the IFNα/γ was released at pH 7.4 in PBS (Figure 5B; ***, *p* < 0.001). On the basis of these release profiles, we speculated that IFNα/γ@PAM^gel^ could deliver more IFNα/γ molecules into the intestinal tract and prevent IFNα/γ leakage in the stomach. This process effectively protects IFNα/γ from enzymatic degradation by pepsin, thereby increasing the bioavailability of IFNα/γ.

### IFNα/γ@PAM^gel^ has no adverse effects on the duodenum

**Figure 6:**
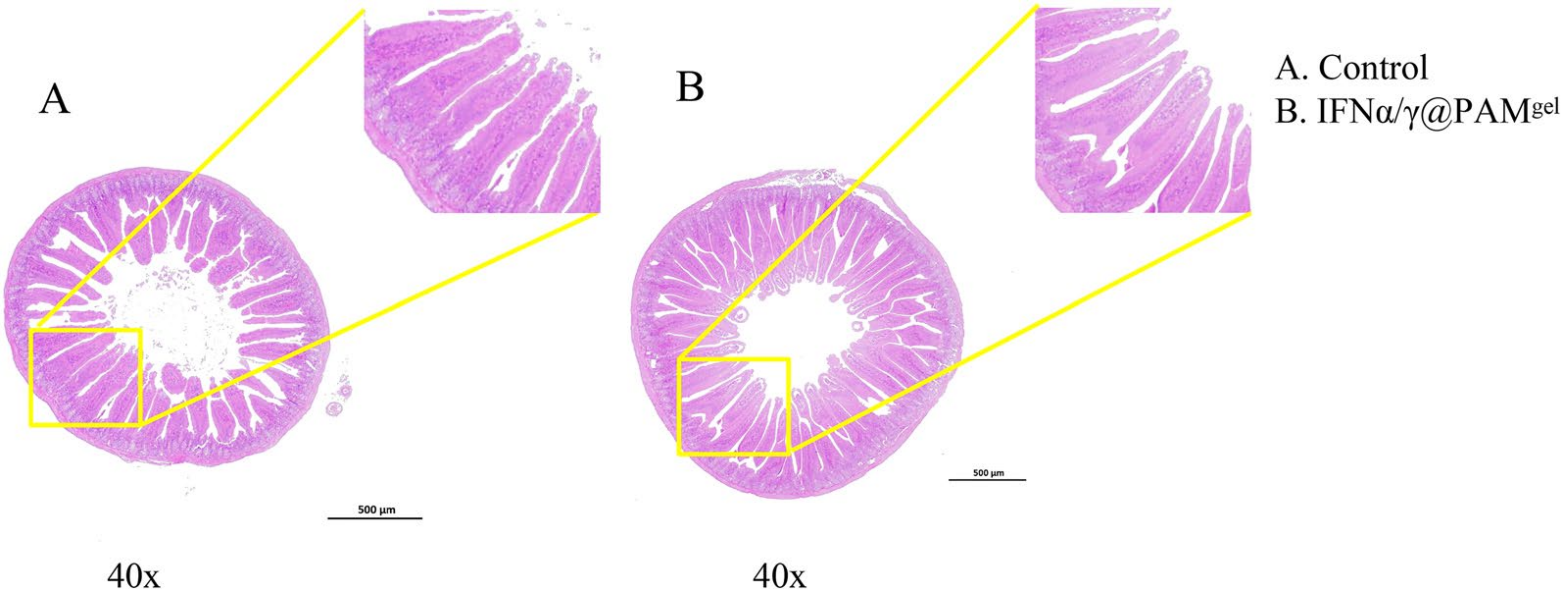
**Pathological changes in mouse duodenal tissue**. Three days after dosing, the toxicity of IFNα/γ@PAM^gel^ was evaluated according to the degree of pathological damage to the mouse duodenal tissue. **A** Histopathological observation of haematoxylin‒eosin (H&E)-stained sections from the control group. **B** Histopathological observation of haematoxylin‒eosin (H&E)-stained sections from the IFNα/γ@PAM^gel^ group.

The duodenum is the main absorption site in the body of an animal. Damage to the intestinal villi or epithelium severely affects the absorption of substances. To evaluate the safety of IFNα/γ@PAM^gel^ in the duodenum, the mice were orally administered IFNα/γ@PAM^gel^ for three consecutive days. The toxicity of IFNα/γ@PAM^gel^ was then evaluated on the basis of the degree of pathological damage to the duodenum tissue of the mice. Histopathological examination of the duodenums of the mice in both the oral IFNα/γ@PAM^gel^ and PBS groups revealed no evidence of pathological damage (Figures 6A and B). Moreover, the density and length of the intestinal villi were essentially the same in the IFNα/γ@PAM^gel^ and PBS groups. Thus, the safety of IFNα/γ@PAM^gel^ in the duodenum of mice has been established, confirming its suitability as a secure delivery vector for oral drug administration systems.

### Orally administered IFNα/γ@PAM^gel^ promotes the proliferation of CD4^+^ and CD8^+^ T lymphocytes in vivo

**Figure 7:**
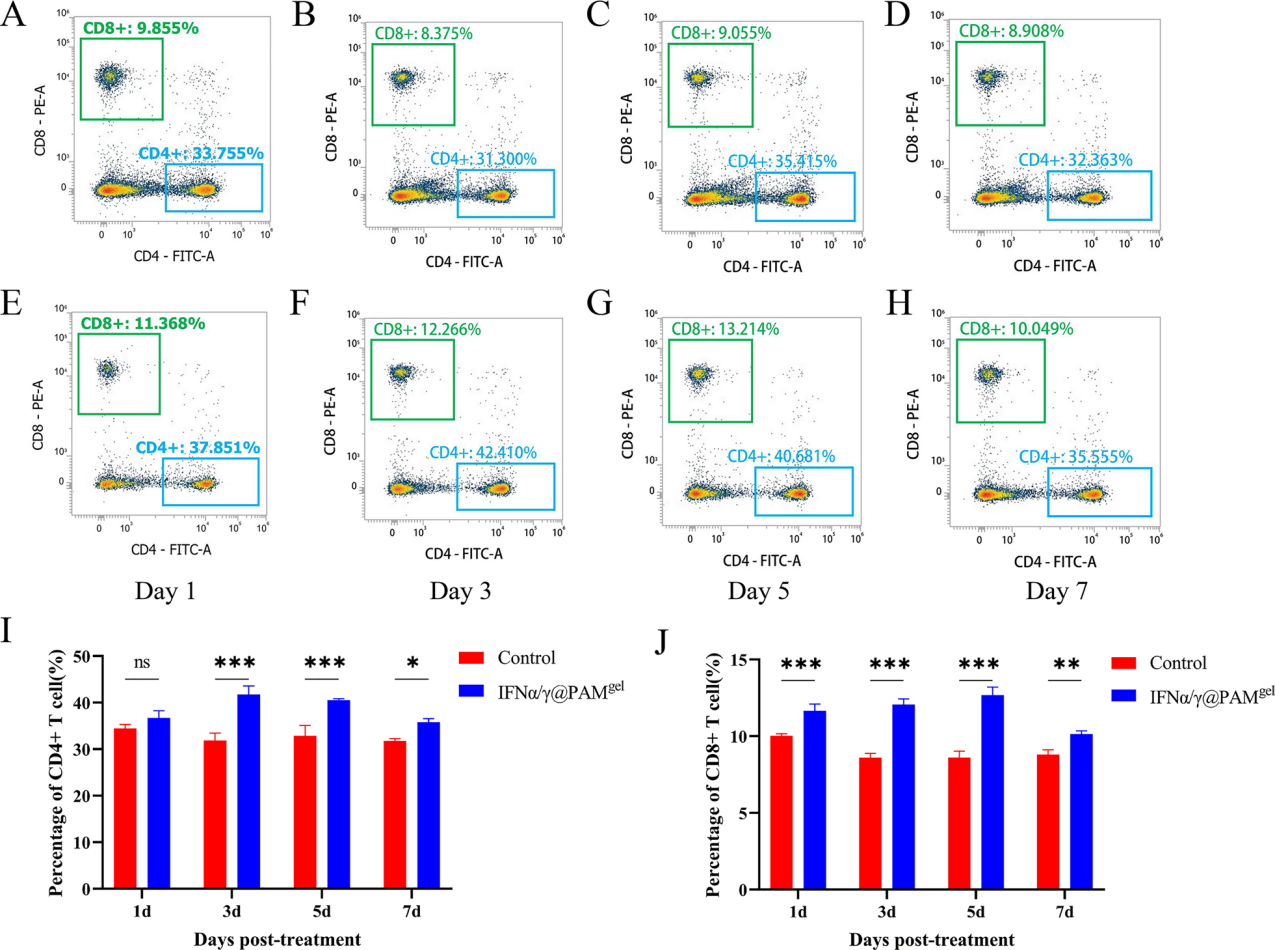
**Analysis of CD**_4_^+^** and CD**_8_^+^** T cells in the blood of experimental mice.** **A**–**H** Changes in CD4^+^ and CD8^+^ T-cell levels on day 1, 3, 5, and 7 in mice in the oral IFNα/γ@PAM^gel^ group and control group. **I**–**J** Ratios of CD4^+^ and CD8^+^ T cells. **I** CD_4_^+^ levels showed no difference after one day of oral administration between IFNα/γ@PAM^gel^ and the PBS control groups but showed a significant difference 3 to 7 days after administration. (*p < 0.05; ***p < 0.001; ns, not significant). **J** There was a significant increase in the ratio of CD8^+^ T cells in the IFNα/γ@PAM.^gel^ group compared with that in the control group from 1 to 7 days. (**p < 0.01; ***p < 0.001).

IFNγ has immunomodulatory effects, and consequently, the effects of IFNα/γ@PAM^gel^ on the proliferation of CD4^+^ and CD8^+^ T lymphocytes in peripheral blood were investigated by FCM. The results revealed that the numbers of CD4^+^ and CD8^+^ T lymphocytes were significantly greater in the IFNα/γ@PAM^gel^ group (Figures 7E–H) than in the PBS group (Figures 7A–D). Furthermore, there was no discernible difference in CD4^+^ levels between the IFNα/γ@PAM^gel^ and PBS groups after one day of oral administration. However, a significant difference was observed at 3 and 5 days post-administration (Figure 7I, *p* < 0.001). Seven days after oral administration, the number of CD4^+^ T lymphocytes in the IFNα/γ@PAM^gel^ oral group decreased but was still greater than that in the PBS group (Figure 7I, p < 0.05). The levels of CD8^+^ T lymphocytes in the IFNα/γ@PAM^gel^ group were significantly different from those in the PBS group throughout the experiment (Figure 7J, *p* < 0.001, *p* < 0.05). These findings suggest that the oral administration of IFNα/γ@PAM^gel^ can increase the proliferation of CD4^+^ and CD8^+^ T lymphocytes in vivo.

### IFNα/γ@PAM^gel^ induces the expression of ISGs in vivo

**Figure 8:**
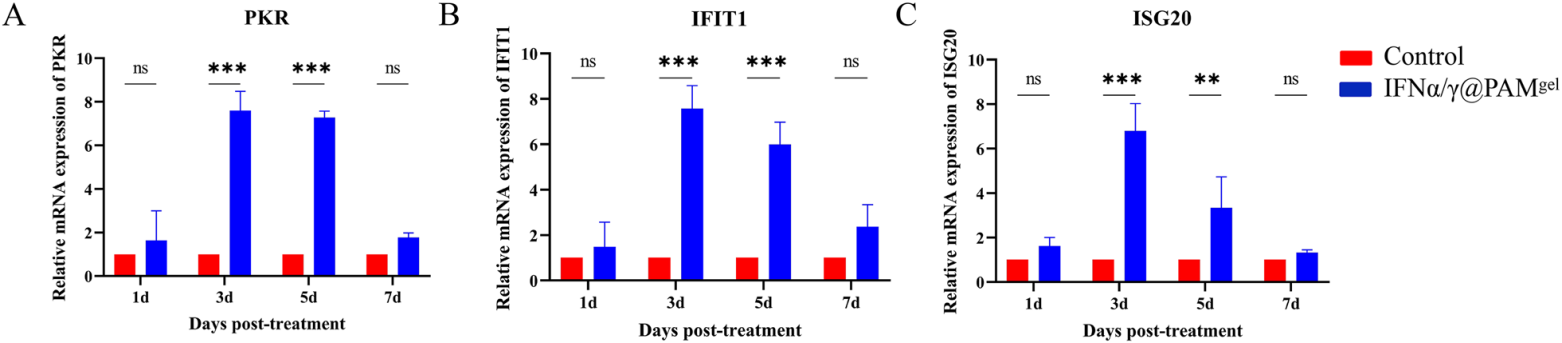
**IFNα/γ@PAM**^gel^**induced ISG (Pkr, Ifit1, and Isg20) expression in mice**. The spleens were collected at the indicated time points. RT‒qPCR analysis of *Pkr* (A), *Ifit1* (**B**), and *Isg20* (**C**) mRNA in mice from the fresh spleen samples of the IFNα/γ@PAM^gel^ and control groups collected on days 1, 3, 5, and 7. Mice in the treatment group were administered IFNα/γ@PAM^gel^ orally for three days, and the controls were untreated. Total RNA from the spleen tissue was extracted using RNA Plus reagent and reverse transcribed into cDNA. Relative gene expression analysis was performed using the 2.^−ΔΔCt^ method, and β-actin was used as an endogenous control. The data are representative of three independent experiments analysed by Student’s *t* tests (** p < 0.01; *** p < 0.001; ns, not significant).

To further validate the impact of the oral administration of IFNα/γ@PAM^gel^ on the innate immune response in the host, we extracted mRNA samples from mouse spleens to perform qRT-PCR analysis of three ISGs (*Pkr*, *Ifit1*, and *Isg20*). The results revealed that the ISG levels in the IFNα/γ@PAM^gel^ treatment group were significantly greater than those in the PBS group at 3 and 5 days post-treatment (Figures 8A–C, *p* < 0.001). The ISG mRNA upregulation peaked three days post-treatment at approximately eightfold, with a significant difference compared with that in the control group (*p* < 0.001). Up to 7 days post-treatment, the mRNA levels of the different ISGs in the IFNα/γ@PAM^gel^ treatment group were still greater than those in the control group, albeit not significantly. The findings of this study demonstrate that the oral administration of IFNα/γ@PAM^gel^ elicits in vivo induction of ISG expression.

### IFNα/γ@PAM^gel^ inhibits PRV infection and decreases the tissue viral load in vivo

**Figure 9:**
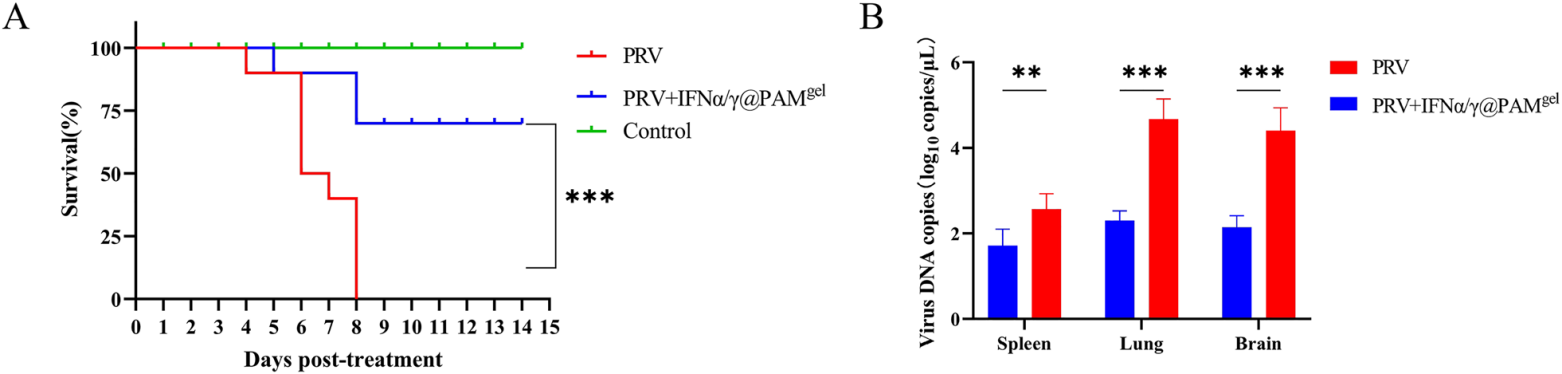
**Protective efficacy of IFNα/γ@PAM**^gel^** against PRV infection in mice**. **A** Survival rates of mice after challenge. **B** Viral DNA copies in different tissues (spleen, lung, and brain). Viral DNA copies were quantified for each tissue using qPCR and calculated as viral DNA copies per microliter. The data are presented as the mean ± standard deviation from three experiments. The statistical significance of differences between IFNα/γ@PAM^gel^-treated and untreated groups was determined using Student’s *t* tests (**p < 0.01; ***p < 0.001; ns, not significant).

To further assess the therapeutic efficacy of oral IFNα/γ@PAM^gel^ against viral infections, we employed PRV-infected mice as an experimental model for treatment. PRV-infected mice in the untreated group presented characteristic clinical symptoms, including pruritus, scratching, gnawing of the facial skin, skin lesions accompanied by tissue damage and bleeding, and neurological symptoms (data not shown). The mice in the untreated group started dying at 4 dpt, and all the mice had died by 8 dpt (Figure 9A). The mice in the oral IFNα/γ@PAM^gel^ treatment group presented milder clinical symptoms than did those in the untreated group. The mice that received oral IFNα/γ@PAM^gel^ exhibited mortality starting at 5 dpt, with a recorded survival rate of 70% (Figure 9A). All the mice in the control group survived and appeared to be in a good mental state. To further investigate the impact of IFNα/γ@PAM^gel^ on PRV replication in vivo, we quantified the viral DNA copy number in various tissues (spleen, lung, and brain) via qPCR. The results revealed a significant reduction in the PRV DNA copy number in the spleen, brain, and lung tissue of the IFNα/γ@PAM^gel^-treated group compared with that in the untreated group, with a decrease of approximately 2.0 log units. (Figure 9B, *p* < 0.01 and *p* < 0.001). The findings of this study suggest that the use of hydrogels as carriers for recombinant IFNα/γ protein has potential for ameliorating clinical symptoms, preventing PRV-related mortality, and effectively suppressing PRV replication in diverse tissues and organs.

### IFNα/γ@PAM^gel^ reduces PRV-induced pathological damage

**Figure 10:**
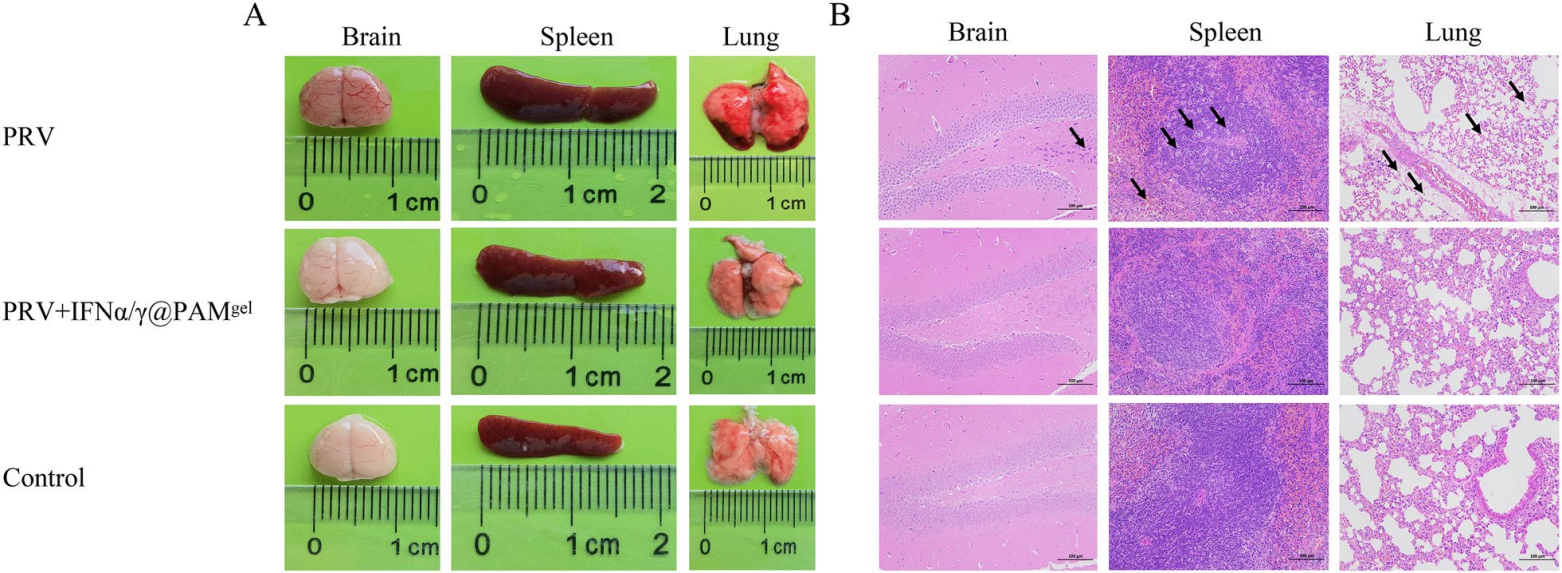
**Pathological changes in mouse organs (brain, spleen, and lung)**. **A** Representative photographs of the appearance of the whole organs of mice from the PRV, PRV + IFNα/γ@PAM^gel^, and control groups. **B** Histopathological observation of hematoxylin–eosin (H&E)-stained brain, spleen, and lung sections from each group of mice. PRV, pseudorabies virus-infected; PRV + IFNα/γ@PAM^gel^, pseudorabies virus-infected and orally administered humic acid hydrogel loaded with interferon α and γ fusion protein.

PRV infection can cause tissue and organ damage. To evaluate this, tissue samples from the lungs, spleen, and brain of mice in different experimental groups were collected for comparative analysis. The findings revealed that mice infected with PRV in the untreated group presented pronounced hemorrhaging in their brains, spleens, and lungs. The brain, spleen, and lung tissue samples analysed from the mice in the IFNα/γ@PAM^gel^ treatment group exhibited characteristics comparable to those of the control group and did not exhibit evident pathological damage (Figure 10A). In addition, the histopathological results revealed that the brain pyramidal cells were wrinkled and that the boundary between the nucleus and cytoplasm was indistinct in the untreated group. Furthermore, the spleen and lung tissue samples in the untreated group exhibited extensive congestion, accompanied by perivascular edema and loose arrangement of the connective tissue; the spleen also exhibited lymphocyte necrosis and dilation of the medullary sinus. In the treatment group, only minor local hemorrhaging and alveolar wall thickening were occasionally observed (Figure 10B). The control group exhibited intact tissue structures with distinct boundaries. These findings demonstrate the protective effect of the oral administration of IFNα/γ@PAM^gel^ against PRV-induced pathological damage in infected mice.

## Discussion

Here, we present an effective potential treatment with antiviral and immunomodulatory effects on PRV. This treatment uses a hydrogel as a delivery carrier of IFN to produce an antiviral effect via oral administration. Using hydrogels as an oral drug delivery system is one method to protect protein drugs from destruction by stomach acid [54, 55]. Our experiments demonstrated that the fusion protein rPoIFNα/γ can inhibit PRV replication in vitro. Furthermore, the prepared hydrogel carrier performed excellently in in vitro experiments. To comprehensively evaluate the prophylactic and therapeutic effects of IFNα/γ@PAM^gel^ on pseudorabies in mice, we investigated the clinical, pathological, viral load, and immunological changes in mice infected with PRV after treatment with and without IFNα/γ@PAM^gel^. Our results showed that IFNα/γ@PAM^gel^ induces ISG expression in mice. The clinical symptoms of pseudorabies and tissue damage caused by PRV infection were also alleviated.

Previous studies have focused primarily on individual IFN family members, including IFNα, IFNλ, IFNγ, and IFNδ [41, 56–58]. Partial mixtures of IFNs from different family members can exert synergistic effects, but not all of them can [36]. Additionally, the production process is further complicated by the requirement of expressing two or more proteins, even if a combination of IFN family members has a synergistic effect. Our previous studies revealed that the expression of IFNα and IL-2 fusion proteins combines their functions and prolongs their effects [59]. These findings reveal the potential of tandem proteins generated through gene fusion to exhibit dual functionalities within a single molecule. On the basis of this premise, we created a recombinant IFNα and IFNγ fusion protein using a flexible linker region, which enabled us to validate its in vivo and in vitro anti-PRV activity. The results showed that the rPoIFNα/γ fusion protein could be expressed and purified and had no obvious toxic effects on cells. In addition, the recombinant protein showed strong antiviral activity against both VSV and PRV (Figure 2D), which was greater than that of monomeric IFN alone (data not shown). Therefore, our strategy of using a fusion protein is feasible. Nevertheless, in our study, the recombinant protein still had certain limitations. For example, the expression of this protein occurs in the form of inclusion bodies, resulting in reduced biological activity compared with that of soluble proteins. In this study, the appropriate concentration of recombinant PoIFNα/γ was tested, and the results revealed that 1.0 g/L was the minimum concentration that completely inhibited PRV in vitro (Additional file 3). In follow-up experiments, we will attempt to improve the antiviral activity of the IFN fusion protein by changing the expression system, optimizing the expression conditions, and reducing the potential adverse effects caused by factors such as dose.

The development of hydrogels dates back to 1960. Since then, hydrogels have been widely used in the field of drug delivery research for slow drug release, especially for oral drug delivery systems [60]. Oral administration is convenient and has little impact on animals, which is convenient for large-scale use. The efficacy of protein drugs is compromised upon their entry into the digestive tract due to their susceptibility to degradation by stomach acid and enzymes, leading to therapeutic failure. To solve this problem, we investigated a pH-sensitive hydrogel that acts as a carrier for IFNα/γ (IFNα/γ@PAM^gel^) and prevents its destruction by stomach acids and enzymes. The synthesized polymeric hydrogel in this study is nontoxic and has a three-dimensional network structure (Figure. 3), enabling efficient absorption and retention of substantial amounts of water or biological fluids. Moreover, the intestine is the main site of nutrient and drug absorption. Therefore, the duodenum was chosen for the safety evaluation. The results revealed that no pathological injury occurred in the duodenal histopathology of the mice in the oral IFNα/γ@PAM^gel^ group (Figure 6B), and we believe that IFNα/γ@PAM^gel^ is safe for the body, which aligns with findings reported in previous studies [61, 62]. Additionally, the synthesized hydrogel exhibited pronounced pH-responsive characteristics (Figure 5A), with notably superior expansion performance observed under slightly alkaline conditions compared with that observed under acidic conditions [45, 55]. The release profile also demonstrated that the prepared hydrogel exhibited pH-dependent control over IFNα/γ release (Figure 5B), thereby enhancing the bioactivity of orally administered IFNα/γ [54]. The absorption bands of the cargo-free PAM^gel^ (Figure 4A) at 3420 cm^−1^ and 1645 cm^−1^ in the FT-IR spectra may be caused by the stretching vibrations of -OH and C = O, respectively [63, 64]. The latter observed peak typically corresponds to the amide I C = O stretching vibration, which is commonly found in the spectral range of 1640–1650 cm^−1^ [65]. The observed vibrational peaks in the amide II region for IFNα/γ and IFNα/γ@PAM^gel^ are consistent with the documented natural helical conformation and protein aggregation values previously reported [66]. The swelling behavior of hydrogels plays a pivotal role in biological applications because of its profound impact on diffusion, fluid dynamics, and surface characteristics. The present study successfully demonstrated various characteristics of the hydrogel, which aligns with the findings of previous researchers, who have emphasized the universal applicability of hydrogel carriers [67]. In future studies, we will further investigate strategies to increase the palatability of the hydrogel, prolong its residence time in the duodenum, and extend the duration of IFN action.

The cellular immune response plays a pivotal role in the eradication of viruses and confers protection against viral infections. Although humic acid has a positive effect on animal immune regulation, the effect is limited. Studies have shown that adding humus to chicken diets does not lead to an increase in the proportion of T lymphocytes [68, 69]. However, our results revealed increases in both the CD4 + and CD8 + T lymphocyte proportions. Therefore, we further speculate that this effect is caused by rPoIFNα/γ. In this study, FCM analysis revealed a significant increase in CD4^+^ T lymphocytes following the oral administration of IFNα/γ@PAM^gel^ (Figure 7I). Various cytokines can induce the differentiation of CD4^+^ T cells into distinct subsets of regulatory T cells, including Th1, Th2, and Th17 cells [70]. Moreover, Th1 cells secrete cytokines, including IFNγ and tumor necrosis factor-α, which can stimulate macrophage activation and delay hypersensitivity reactions [71, 72]. Moreover, IFNα/γ@PAM^gel^ increased the number of CD8^+^ T cells (Figure 7J), which are known to play a crucial role in viral clearance [73]. These findings suggest that IFNα/γ@PAM^gel^ enhances the proliferation of Tc and Th cells in mice. Consequently, we hypothesize that the primary mechanism by which IFNα/γ@PAM^gel^ exerts its protective effect is through the induction of a robust cellular immune response. The IFN family effectively establishes an antiviral barrier by orchestrating the induction of numerous ISGs and antiviral genes [74]. At present, we have not found a suitable method for real-time detection of IFN release in various tissues. Considering that IFN exerts its antiviral effect mainly by stimulating cells to produce ISGs and that the expression level of ISGs is positively correlated with the content of IFN, we indirectly reflect the release of IFN through changes in the expression level of ISGs. Our findings suggest that the administration of IFNα/γ@PAM^gel^ can elicit increased expression of PKR, IFIT1, and other antiviral mediators in vivo (Figure 8), which aligns with previous investigations [36, 63]. Therefore, we infer that the release of recombinant PoIFN-α/γ in different tissues was greatest at 3–5 days after treatment. Therefore, this may be the primary reason for the increased survival rate and reduced viral load in the IFNα/γ@PAM^gel^ treatment group (Figure 9A and B). Studies have shown that beneficial systemic effects against different infectious diseases can be achieved when IFNs derived from chickens, mice, cows, sheep, or humans are administered orally [75]. Furthermore, chickens that received chicken IFN-α orally presented more favourable therapeutic outcomes than did those that received it intramuscularly [76]. In this study, oral administration of IFNα/γ@PAM^gel^ also significantly reduced clinical symptoms and tissue damage in PRV-infected mice (Figures 10A and B). This evidence provides a theoretical basis for oral IFN administration. Our future research will focus on determining the optimal dosage of rPoIFN and enhancing its oral bioavailability.

Overall, a fusion IFNα/γ protein that combines the antiviral functions of IFNα and the immunoregulatory functions of IFNγ was expressed. A pH-responsive hydrogel loaded with IFNα/γ was formulated to withstand the acidic gastric environment and enable controlled release and absorption in the small intestine to facilitate oral administration. The use of this method ameliorated the clinical symptoms exhibited by mice infected with PRV and significantly mitigated their mortality rate. Our findings provide improved solutions for treating PRV and contribute to future research on protein-based drugs. Most importantly, the results indicate that it may be possible to administer protein-based drugs orally.

## Supplementary Information

**Additional file 1: rPoIFN α/γ fusion gene sequence**.

**Additional file 2: Hydrogel porosity. The results of the porosity experiment indicate that the hydrogel has a porosity of approximately 60%.**

**Additional file 3: Experiments on concentration gradients.**

## Data Availability

The data supporting the conclusions of this article are included within the article. The raw data are available upon request.
