# Supplementary material for: A pH-triggered self-releasing humic acid hydrogel loaded with porcine interferon α/γ achieves anti-pseudorabies virus effects by oral administration
Source: Vet Res. 2024 Nov 20;55:153. doi: 10.1186/s13567-024-01411-w (PMC11580204; doi:10.1186/s13567-024-01411-w)
Supplement: Supplementary file 1 — Additional file 1: rPoIFN α/γ fusion gene sequence. [file 13567_2024_1411_MOESM1_ESM.docx]

**Additional file 1 rPoIFN α/γ fusion gene sequence.**

GAATTCTGTGATCTGCCGCAGACCCACAGCCTGGCGCACACCCGTGCTCTGCGTCTGCTGGCGCAGATGCGTCGTATCTCTCCGTTCAGCTGCCTGGATCATCGTCGTGATTTCGGTAGCCCGCACGAAGCCTTCGGTGGTAACCAGGTTCAGAAAGCGCAGGCGATGGCGCTGGTTCACGAAATGCTGCAGCAGACCTTCCAGCTGTTCAGCACCGAAGGCTCTGCGGCAGCGTGGAACGAAAGCCTGCTGCATCAGTTCTGCACCGGTCTGGATCAGCAGCTGCGTGACCTGGAAGCGTGCGTTATGCAGGAAGCGGGCCTGGAAGGCACCCCGCTGCTGGAAGAAGATTCTATCCTGGCGGTTCGTAAATACTTCCACCGTCTGACCCTGTACCTGCAGGAAAAATCTTACAGCCCGTGCGCGTGGGAAATCGTTCGTGCTGAAGTTATGCGTAGCTTCTCTAGCAGCCGTAACCTGCAGGATCGTCTGCGTAAAAAAGAAGGTGGTGGTGGTAGCGGCGGCGGCGGTTCCGGCGGCGGCGGTTCTCAGGCGCCGTTCTTCAAAGAAATCACCATCCTGAAAGATTACTTCAACGCATCTACCAGCGATGTTCCGAACGGTGGTCCGCTGTTCCTGGAAATCCTGAAAAACTGGAAAGAAGAATCTGATAAGAAAATCATCCAGTCTCAGATCGTTAGCTTCTACTTCAAATTCTTCGAAATTTTCAAAGATAACCAGGCGATCCAGCGTTCTATGGATGTTATCAAACAGGATATGTTCCAGCGTTTCCTGAACGGTAGCTCCGGTAAACTGAACGATTTCGAAAAACTGATCAAAATCCCGGTTGATAACCTGCAGATCCAGCGTAAAGCAATCTCTGAACTGATCAAAGTTATGAACGATCTGAGCCCGCGTAGCAACCTGCGTAAACGTAAACGTAGCCAGACCATGTTCCAGGGTCAGCGTGCGAGCAAATAACTCGAG
