# Supplementary material for: A pH-triggered self-releasing humic acid hydrogel loaded with porcine interferon α/γ achieves anti-pseudorabies virus effects by oral administration
Source: Vet Res. 2024 Nov 20;55:153. doi: 10.1186/s13567-024-01411-w (PMC11580204; doi:10.1186/s13567-024-01411-w)
Supplement: Supplementary file 2 — Additional file 2: Hydrogel porosity. The results of the porosity experiment indicate that the hydrogel has a porosity of approximately 60%. [file 13567_2024_1411_MOESM2_ESM.docx]

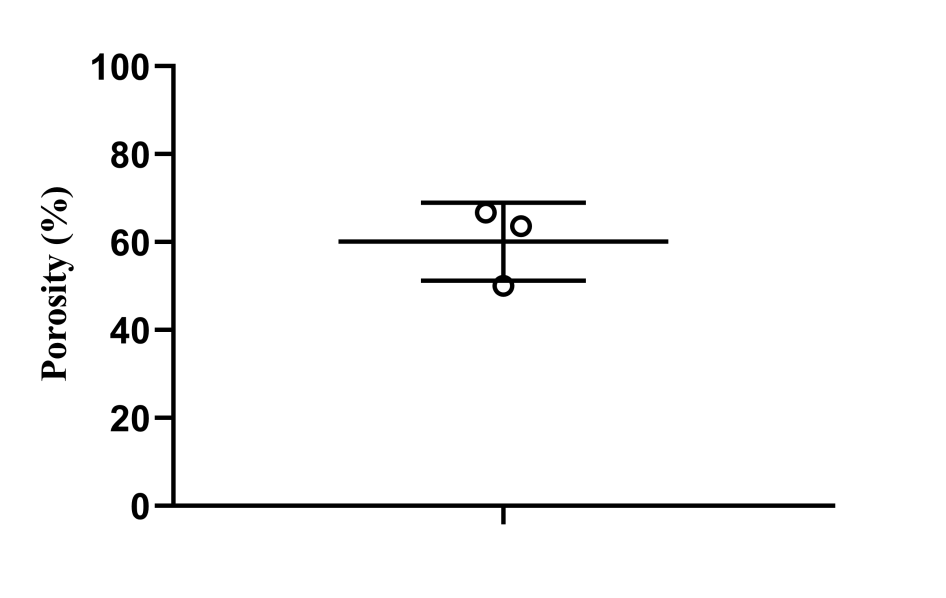


**Additional file 2** **The results of the porosity experiment show that the hydrogel has a porosity of about 60% and can adsorb more drugs**. It is a good oral drug carrier.
