# Supplementary material for: A pH-triggered self-releasing humic acid hydrogel loaded with porcine interferon α/γ achieves anti-pseudorabies virus effects by oral administration
Source: Vet Res. 2024 Nov 20;55:153. doi: 10.1186/s13567-024-01411-w (PMC11580204; doi:10.1186/s13567-024-01411-w)
Supplement: Supplementary file 3 — Additional file 3: Experiments on concentration gradients. [file 13567_2024_1411_MOESM3_ESM.docx]

| IFN concentration gradient | CPE Hole | No CPE Hole | Percentage of CPE |
| --- | --- | --- | --- |
| 2.5g/L | 0 | 8 | 0% |
| 2.0g/L | 0 | 8 | 0% |
| 1.0g/L | 0 | 8 | 0% |
| 0.5g/L | 2 | 6 | 25% |
| 0.25g/L | 5 | 3 | 62.5% |
| 0g//L | 8 | 0 | 100% |

**Additional file 3** **Experiments on concentration gradients.**
